# Overcoming Challenges in Hemihyperplasia Through Surgical Innovation and Genetic Diagnosis: A Case Report

**DOI:** 10.7759/cureus.54445

**Published:** 2024-02-19

**Authors:** Bhagyesh Sapkale, Raju K Shinde, Umesh Kakde

**Affiliations:** 1 Medicine, Jawaharlal Nehru Medical College, Datta Meghe Institute of Higher Education and Research, Wardha, IND; 2 General Surgery, Jawaharlal Nehru Medical College, Datta Meghe Institute of Higher Education and Research, Wardha, IND

**Keywords:** multidisciplinary approach, genetic counseling, medical genetics, surgical intervention, pediatric surgery, challenges, case report, genetic diagnosis, surgical innovation, hemihyperplasia

## Abstract

This case report sheds light on the complex management of hemihyperplasia (HHP), highlighting the difficulties associated with diagnosis and the critical importance of a multimodal approach to treatment. The story of Acharya Vinoba Bhave Rural Hospital's (AVBRH) successful resolution following a misdiagnosis at another clinic emphasizes the value of expert care. The successful outcome resulted from the fusion of surgical innovation, genetic insights, and psychosocial factors through genetic testing, liposuction, and postoperative rehabilitation. This example emphasizes the need to treat congenital illnesses holistically and the transforming power of individualized, multidisciplinary treatment to improve the functional and esthetic elements of life for patients with HHP.

## Introduction

When a person has hemihyperplasia (HHP), one side of their body or a portion of one is bigger than the other side to the degree that it is thought to be larger than usual [1]. A clinician should be able to detect the asymmetry "from the end of the bed" because it is challenging to construct a set of clinical criteria for diagnosing HHP [2]. HHP was previously known as hemihypertrophy. Numerous congenital syndromes, such as Silver-Russell and Beckwith-Wiedemann, exhibit HHP [3,4]. Asymmetry in HHP might be minor or severe [5]. Hepatoblastoma and Wilms's tumour are the two central embryonal tumours for which HHP is linked to an elevated risk [6,7]. A tumour screening protocol is advised in kids having isolated HHP and Beckwith-Wiedemann syndrome due to the increased tumour risk [3,7]. Children with HHP may occasionally have varying leg lengths [8], and they may also experience a curvature of the spine called scoliosis [9].

## Case presentation

A 19-year-old male presented with congenital right-hand hypertrophy at another clinic in 2016. Despite seeking medical attention, the clinician could not diagnose the condition accurately and recommended exercise as the sole intervention to address the hypertrophy. The clinician dismissed the need for medication, attributing the hypertrophy to an unexplained swelling. In agreement with the clinician, the patient attempted to address the issue through exercise, but unfortunately, this approach proved ineffective. The patient came to Acharya Vinoba Bhave Rural Hospital (AVBRH) for further proper diagnosis and was very dissatisfied with the previous clinician's approach in 2022.

The patient was examined by an orthopaedic specialist, and it was determined that the right hand of this patient was more significant than the other hand. Asymmetry in the size of both hands was thus seen. A more significant swelling and bulk were observed in this hand. Fatty tissue accumulated in the right hand in both the arm and forearm regions. There was abnormal growth of muscles and bones in the right hand. Asymmetric joint development resulted in variations in the length of limbs. There were functional restrictions related to the right hand. After identifying the aforementioned clinical characteristics frequently linked to HHP, a confirmatory histology analysis was conducted. X-rays were taken to observe bone and tissue abnormalities, and genetic testing results described a mutation in the PIK3CA gene that confirms HHP. Finally, it was concluded that he had HHP after this diagnostic evaluation. Pre-surgical preparation and intervention plan for optimal surgical outcomes are described in Table 1.

**Table 1:** Pre-surgical preparation and intervention plan for optimal surgical outcomes

Stage	Intervention	Rationale
Pre-consultation	Medical history review, physical examination, blood tests, X-rays	Assess overall health, confirm the diagnosis, and evaluate risks and suitability for surgery
Preoperative education	Explanation of potential complications and recovery process	Prepare the patient for what to expect after surgery
Medication management	Review and adjust any existing medications and prescribe antibiotics/pain management medications	Prevent infection and manage pain postoperatively
Dietary counselling	Specific dietary instructions to optimize pre-surgical nutrition and wound healing	Improve surgical outcome and recovery
Stop smoking/alcohol consumption	Discontinuation of smoking and alcohol helps with wound healing and reduces surgical risks	Enhance recovery and minimize complications

Clinical findings 

**Figure 1:**
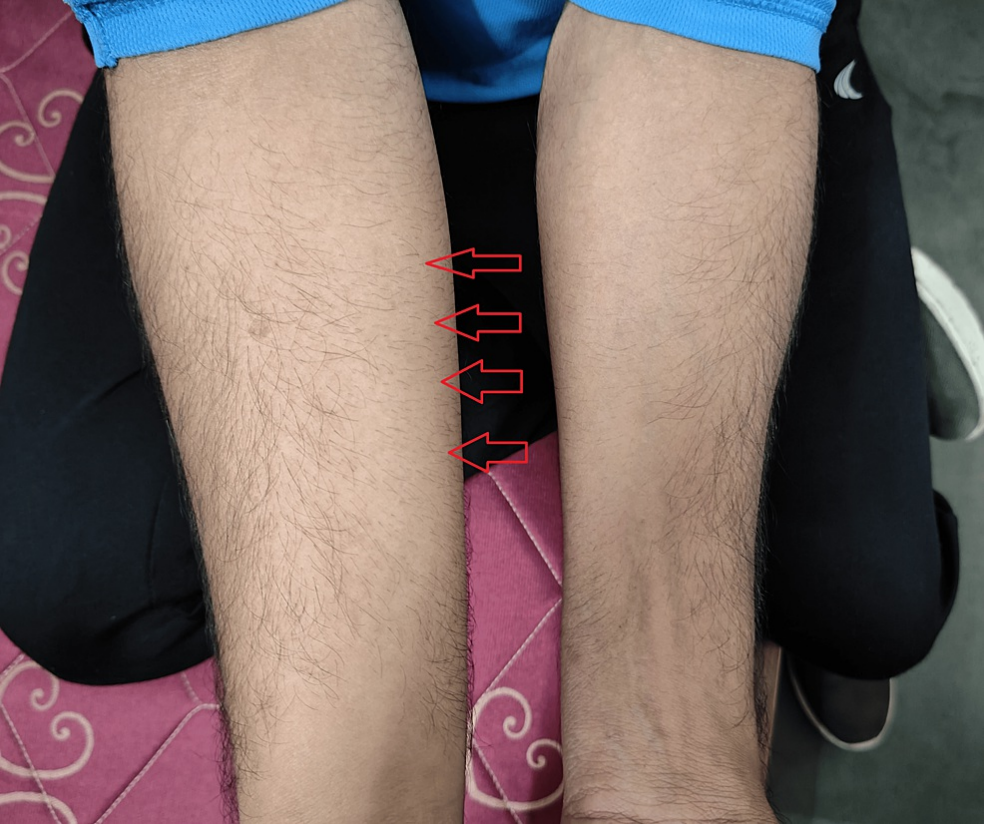
The pre-operation condition of the patient Image Credit: Author

A significant right-hand bulk from birth featured the clinical findings of this 19-year-old patient. The bulk and volume of soft tissues in the right arm were more significant than in the left, exhibiting a solid asymmetry. This hypertrophy affected the forearm and upper arm, extending from the shoulder to the fingertips. A functional impairment affects day-to-day activities in the patient's right hand. The condition was misdiagnosed at another clinic, which made it difficult to determine a suitable treatment strategy for the patient's right-hand hypertrophy. It was probably caused by the condition's rarity, complexity, and lack of awareness, in addition to the requirement for comprehensive diagnostic evaluations. The orthopaedic specialist's assessment at AVBRH in 2022 validated the hypertrophy of soft tissues in the right hand. Imaging studies, which include X-rays, are used to determine the degree of bone involvement and soft tissue abnormalities. The patient's general health, the localized nature of the hypertrophy, and the desire for a focused fat reduction all played a significant role in pursuing a minimally invasive procedure, liposuction. The effective liposuction treatment resolved the patient's cosmetic and functional issues, decreasing the extra adipose tissue. Symptoms and treatment aspects of HHP are described in Table 2. The pre-operation condition of the patient is shown in Figure 1, showing right-hand hypertrophy. The timeline of events in the case is shown in Table 3.

**Table 2:** Symptoms and treatment aspects in hemihyperplasia AVBRH: Acharya Vinoba Bhave Rural Hospital

Symptom	Treatment aspect
Significant right-hand bulk from birth	Minimally invasive liposuction to reduce adipose tissue
Asymmetry in soft tissue volume between right and left arms	Liposuction to address hypertrophy and improve symmetry
Functional impairment in the right hand affecting daily activities	Liposuction to improve hand function and regain full use
Difficulty finding a treatment plan initially	Possible causes: rarity, complexity of the condition, and lack of awareness among medical professionals and requirement for comprehensive diagnostic evaluations
Hypertrophy of soft tissues in the right hand	Validated by the orthopaedic specialist at AVBRH in 2022
X-rays determine the degree of bone involvement and soft tissue abnormalities	Imaging studies to aid in diagnosis and treatment planning
Patient's general health, localized nature of hypertrophy, and desire for focused fat reduction	Factors favouring minimally invasive liposuction
Effective liposuction treatment resolving cosmetic and functional issues	Decreased extra adipose tissue and improved hand function and appearance

**Table 3:** The timeline of events AVBRH: Acharya Vinoba Bhave Rural Hospital

Year	Event
2004	Patient's birth with evident right-hand hypertrophy
2016	The patient visited another clinic to seek medical attention
2016-2022	Ongoing challenges and unsuccessful treatment at another clinic
2022	The patient sought care at AVBRH
2022	Evaluation by the AVBRH orthopaedic specialist confirmed the hemihyperplasia diagnosis
2022	Initiation of a comprehensive treatment plan, including the consideration of liposuction
2023	The successful completion of the liposuction procedure at AVBRH resulted in reduced hypertrophy
2023	Postoperative care and rehabilitation to optimize functional outcomes
2023	A positive outcome was observed, marking the successful resolution of the hemihyperplasia

Management of HHP

The treatment strategy was centred on surgical intervention, postoperative care, and precise diagnosis.

Diagnostic Evaluation

The diagnostic evaluation comprised clinical examination evaluating the right hand's asymmetry, soft tissue hypertrophy, and functional restrictions and imaging studies like X-rays and potentially more sophisticated imaging techniques to assess soft tissue anomalies and bone involvement. Since HHP is a congenital condition, genetic testing has been conducted to find related mutations.

Approach to Treatment

Liposuction is a minimally invasive procedure that targets localized fat deposits, so it was chosen as the primary intervention. The instrument used to perform the liposuction is VASERlipo®, manufactured by VASER® from the United States. VASERlipo® is an ultrasound-assisted, minimally invasive liposuction procedure that is gentle and powerful enough to remove fat from larger parts of the body and is utilized as an alternative to the harsh techniques of traditional liposuction. Visible fat is instantly removed using VASERlipo®. It's an advancement over non-invasive techniques that depend on side effects to eliminate modest amounts of fat gradually. In a single surgery, VASERlipo® can sculpt and shape a number of locations, such as the neck and chin, the arms, the back, the buttocks or thighs, the knees, and the ankles. Compared to standard liposuction, VASERlipo® offers smoother shapes with less pain and recovery time because of its unique ability to target fat while sparing other vital tissues. Before the procedure, the patient was administered with local anaesthesia to ensure comfort and pain control during the surgery.

**Figure 2:**
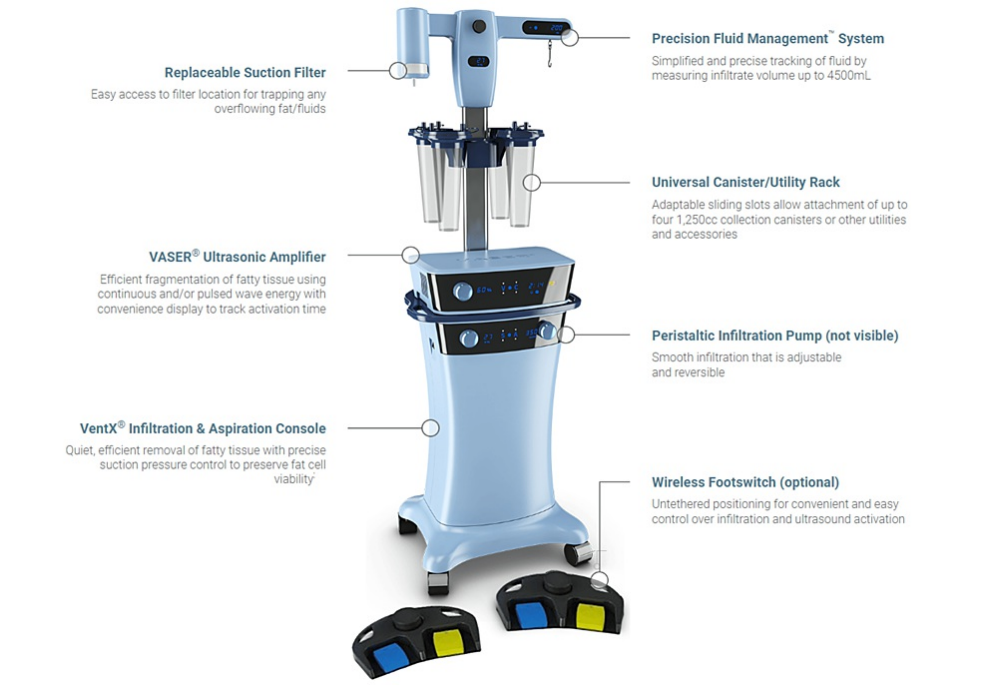
VASERlipo® used for liposuction Image Credit: https://www.vaser.com/hcp/#the-vaser-system VASER® is a precision ultrasound-assisted method that combines mechanical and acoustic fat fragmentation/emulsification. Its goal is to maximize operation speed and efficiency while minimizing tissue harm

The first step during the surgery was infiltration; tumescent fluid containing saline, local anaesthetic, epinephrine, and small gas bubbles 5-10 microns in size was infused throughout the targeted fat tissue in the patient's right hand. Gas bubbles cannot permeate and damage connective tissues or blood vessel walls because of tight connections between cells in these tissues. The second step was fragmentation; using an ultrasonic probe to transmit acoustic energy, clusters of undamaged fat cells were dislodged from the tumescent fluid with minimal damage to arteries, nerves, and other tissues. The bubbles quickly formed and burst. Via the highly localized fluid forces created by the grooves on the ultrasonic probe, dislodged fat clusters can be further divided into smaller clumps for use in fat grafting techniques. The third step was aspiration; when suctioning out the mixed fat cell and tumescent fluid emulsion, a small-diameter cannula was used to remove fat, as it causes less damage to arteries, nerves, and the fibrous tissue matrix than second-generation cannulas do. The fourth and last step was retraction, which is tissue remodeling because the loss of adipose tissue during liposuction made room for retraction to take place. The subcutaneous connective tissue matrix was retained, and the superficial fatty layer was "thinned" with minimal stress to tissues, so post-procedure skin retraction was optimal, which allowed the skin to naturally retract and re-drape to the underlying frame throughout the healing process. A postoperative care plan highlights optimizing functional results and eyeing problems. Physiotherapy and other rehabilitation techniques improved the treated limb's range of motion and functional ability. VASERlipo® used for liposuction is shown in Figure 2.

Follow-Up and Results

**Figure 3:**
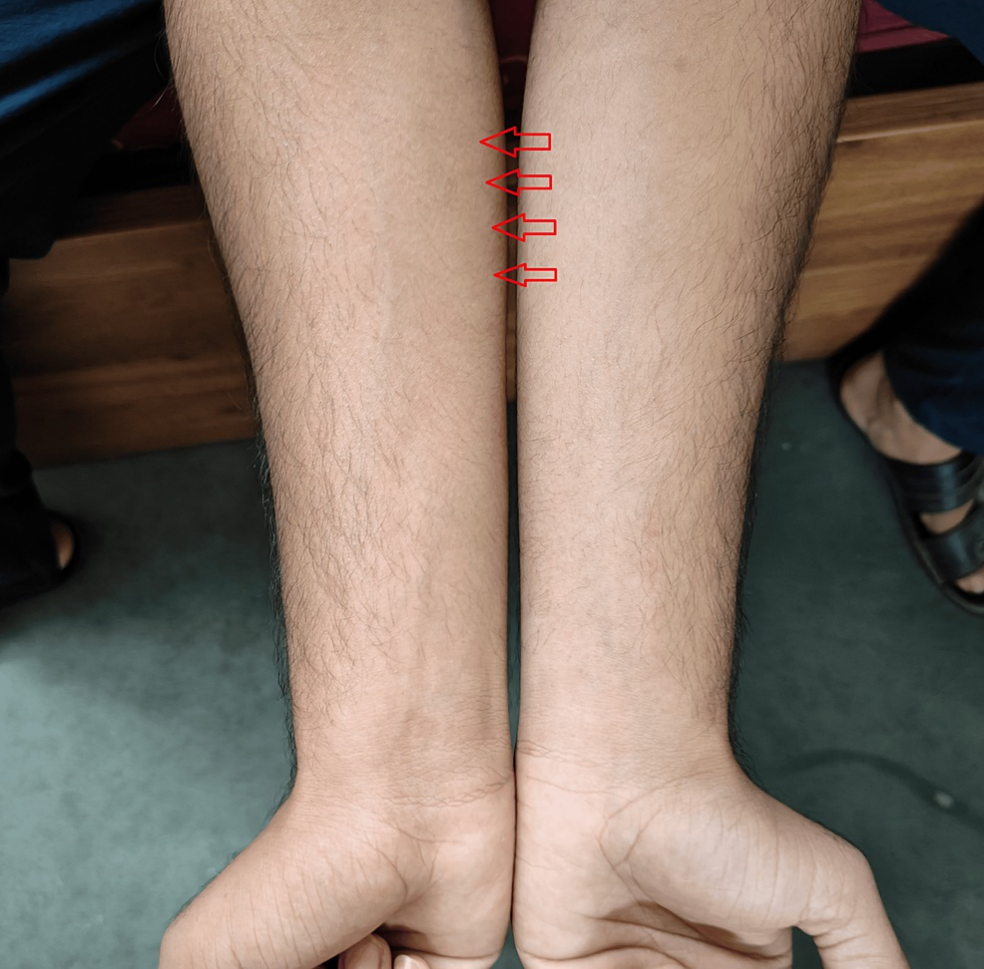
The post-operation condition of the patient Image Credit: Author

After the procedure, the patient underwent a general examination to evaluate any issues and monitor recovery. The patient can now effortlessly move his right hand to perform everyday tasks. Later on, after a follow-up of 12 months, which is enough to declare successful results, no more issues were noticed in the patient. No significant bony changes are observed in the patient as no bones were involved in the relevance to the case. Postoperative care is described in Table 4. The post-operation condition of the patient is shown in Figure 3.

**Table 4:** Postoperative care

Stage	Intervention	Rationale
Immediate recovery	Monitoring vital signs, pain management, and wound dressing changes	Ensure patient safety and monitor for immediate post-surgical complications
Physical therapy	Specific exercises to regain mobility and function in the affected hand	Promote flexibility, strength, and function of the hand and arm
Wound care	Regular cleaning and dressing changes to prevent infection and promote healing	Maintain hygiene and avoid infection at the surgical site
Dietary monitoring	Provide adequate nutrition to support recovery	Optimize healing and prevent nutritional deficiencies
Follow-up appointments	Regular check-ups to monitor progress, address any concerns, and adjust treatment plans as needed	Ensure proper recovery and monitor for long-term complications

## Discussion

This case report underscores the challenges of diagnosing and managing HHP. The initial misdiagnosis at another clinic in 2016 and the subsequent successful resolution at AVBRH in 2022 highlight the importance of expert care and the need for a comprehensive approach to congenital disorders. The rarity and complexity of HHP and a lack of awareness among medical professionals contributed to the initial difficulty in formulating an effective treatment plan. The orthopaedic specialist at AVBRH employed a multidisciplinary approach, combining clinical examination, imaging studies, and genetic testing to confirm the diagnosis. Identifying a mutation in the PIK3CA gene through genetic testing played a crucial role in establishing the diagnosis and guiding the treatment strategy [8,10].

The choice of a minimally invasive procedure, liposuction, as the primary intervention reflects a patient-centred approach, considering factors such as the patient's general health, the localized nature of hypertrophy, and the desire for focused fat reduction [11]. The successful outcome, marked by reduced hypertrophy and improved functionality, highlights the transformative power of individualized, multidisciplinary treatment [5]. The discussion section could be enhanced by emphasizing the clinical significance of this case in advancing our understanding of HHP and its management. Furthermore, acknowledging the evolving role of genetic testing in diagnosing congenital disorders and the potential implications for personalized treatment plans would add depth to the discussion. So, this case report exemplifies a patient's journey with HHP, emphasizing the importance of accurate diagnosis, expert care, and a holistic approach to treatment. The successful resolution at AVBRH is a testament to the transformative impact of individualized, multidisciplinary care in improving the functional and esthetic aspects of life for patients with congenital disorders like HHP.

## Conclusions

This case report highlights the significant hurdles overcome in diagnosing and treating HHP. Misdiagnosis and limited awareness initially proved obstacles, yet AVBRH meticulous approach, encompassing comprehensive diagnostics, tailored interventions, and expert multidisciplinary care, ultimately paved the way for a successful outcome. This case is a powerful example of how accurate diagnosis and personalized treatment strategies can triumph over the intricate challenges associated with rare and complex conditions like HHP.
